# LncRNA LINC00857 regulates the progression and glycolysis in ovarian cancer by modulating the Hippo signaling pathway

**DOI:** 10.1002/cam4.3322

**Published:** 2020-09-12

**Authors:** Xueke Lin, Dilu Feng, Ping Li, Yuchun Lv

**Affiliations:** ^1^ Department of Obstetrics and Gynecology Quanzhou First Hospital Affiliated to Fujian Medical University Quanzhou China; ^2^ Department of Obstetrics and Gynecology Union Hospital Tongji Medical College Huazhong University of Science and Technology Wuhan China

**Keywords:** Hippo signaling pathway, LINC00857, miR‐486‐5p, ovarian cancer, YAP1

## Abstract

Ovarian cancer is one of the most common gynecological cancers with high morbidity and mortality, which seriously endangers women's health and quality of life. Long noncoding RNAs (lncRNAs) can regulate the progression of cancers, including ovarian cancer. LINC00857 (long intergenic non‐protein coding RNA 857) has been discovered to be a crucial factor in the regulation of cancer development. Nevertheless, the specific functions and mechanisms of LINC00857 in ovarian cancer remain unclear. The Hippo signaling pathway can involve in cancer progression. In our research, we aimed to investigate the correlation of LINC00857 and Hippo pathway. Quantitative real‐time polymerase chain reaction assay was utilized to test the expression of LINC00857 in ovarian cancer tissues and cells. Functional experiments revealed that LINC00857 silencing led to the inhibition on cell proliferation, migration, invasion, and glycolysis but accelerated cell apoptosis in ovarian cancer. Mechanism experiments, including RNA immunoprecipitation, RNA pull‐down, and luciferase reporter experiments demonstrated that LINC00857 could regulate YAP1 (Yes1 associated transcriptional regulator) by competitively binding to miR‐486‐5p in ovarian cancer. In a word, this study unveiled that LINC00857 regulates YAP1 by competitively binding to miR‐486‐5p and accelerates ovarian cancer progression.

## Graphical abstract

MiR‐486‐5p restrains cell progression and promotes cell apoptosis in ovarian cancer. Long intergenic non‐protein coding RNA 857 accelerates ovarian cancer progression and glycolysis via regulating Yes1 associated transcriptional regulator.

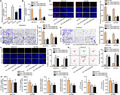

## 1 INTRODUCTION

Ovarian cancer is one of the commonest female malignant tumors, which seriously endangers women's health and quality of life.^1^ It has an increasing incidence and high mortality in recent years.^2^ Since the early symptoms are not obvious, diagnose in the early stage is very difficult. Thus, most of the patients are in the late stage when they are diagnosed,^3^ which leads to the low overall survival rate.^4^ Although current therapeutic strategies have been improved,^5^, ^6^ new biomarkers are still necessary to develop for exploring novel therapeutic targets.

Long noncoding RNAs (lncRNAs) are RNA transcripts longer than 200 nucleotides without the protein‐coding ability.^7^ An increasing number of evidence indicated that lncRNAs are biological participants and modulators in different cellular processes such as proliferation, apoptosis, and migration.^8^ Importantly, lncRNAs are also confirmed to be the crucial factors in assorted cancers and they regulate cancer progression by exerting different functions in cancer cells.^9^ For example, OGFRP1 accelerates cell proliferation and migration of hepatocellular carcinoma through AKT/mTOR and Wnt/β‐catenin signaling pathways.^10^ LINC003121 restrains cell growth of thyroid cancer via the suppression of the PI3K/Akt pathway.^11^ Long noncoding RNA LINC00857 (long intergenic non‐protein coding RNA 857) has been proven to exert a crucial regulatory function in several cancers. For example, LINC00857 exerts the oncogenic role in esophageal adenocarcinoma and knockdown of it represses the progression of esophageal adenocarcinoma.^12^ In addition, LINC00857 was considered to be associated with low survival rate of patients and accelerates tumor progression in lung cancer.^13^ Nevertheless, there are few researche on the role of LINC00857 in ovarian cancer.

The Hippo signaling pathway was primitively found in Drosophila as a modulator of organ size. It could limit cell number by regulating proliferation and apoptosis.^14^, ^15^ YAP1 (Yes1 associated transcriptional regulator) is the crucial downstream oncogene of the Hippo pathway.^16^ In recent years, the Hippo signaling pathway has been found to be closely associated with tumor progression. For example, TNRC6C‐AS1 expedites the methylation of STK4 to inhibit thyroid carcinoma cell apoptosis through the Hippo signaling pathway.^17^ HOX transcript antisense RNA directly binds to SAV1 and activates the Hippo pathway in renal cell carcinoma.^18^

In our research, we focused on the functions of LINC00857 in ovarian cancer and explore the regulatory mechanism between LINC00857 and Hippo pathway.

## 2 MATERIALS AND METHODS

### 2.1 Tissues samples

Fifty ovarian cancer patients who underwent surgery at Quanzhou First Hospital Affiliated to Fujian Medical University from January 2017 to December 2019 were reviewed. Before surgery, all patients did not receive any kind of therapy. After collection, tumor tissues and adjacent non‐tumor tissues were snap frozen in liquid nitrogen and stored at −80°C for RNA extraction and quantitative real‐time polymerase chain reaction (qRT‐PCR) analysis. Clinical samples were collected and analyzed in accordance with the Declaration of Helsinki. All participants or their guardians had provided the written informed consent.

### 2.2 Cell culture

Ovarian cancer cell lines (SKOV3, Caov3, and A2780) and normal human ovarian epithelial cell lines (IOSE‐29) were purchased from ATCC. Cells were cultured in the RPMI‐1640 medium (Gibco) which contained 10% Fetal Bovine Serum (FBS; Gibco) and incubated at 37°C with 5% CO_2_.

### 2.3 Cell transfection

Short hairpin RNAs (shRNAs) targeting LINC00857 (sh‐LINC00857#1, sh‐LINC00857#2) and YAP1 (sh‐YAP1#1, sh‐YAP1#2) were utilized to silence LINC00857 and YAP1. And pcDNA3.1/YAP1 and pcDNA3.1/LINC00857 was utilized for the overexpression of YAP1 and LINC00857. In addition, miR‐486‐5p mimics were utilized for miR‐486‐5p overexpression. These plasmids were purchased from GenePharma. Transfection was performed by utilizing Lipofectamine 3000 (Invitrogen) in line with the user guidance. Each procedure in this experiment was repeated in triplicate. Short hairpin RNA sequences were listed as follows:

sh‐NC: CCGGCATGACTCATCTACATACACTCTCGAG AGTGTATGTAGATGAGTCATGTTTTTG; sh‐LINC00857#1: CCGG TCTACATGCTCATACACTCAACTCGAGTTGAGTGTATGAGCATGTAGATTTTTG; sh‐NC: CCGGTATAGTATCTATCTTACATAT CTCGAGATATGTAAGATAGATACTATATTTTTG; sh‐YAP1#1: CCGGTTATATAGTAAATTTCTCCATCTCGAG ATGGAGAAATTTACTATATAATTTTTG; sh‐YAP1#2: CCGG TCTTTTGATTCTTTAGAGCCACTCGAGTGGCTCTAAAGAATCAAAAGATTTTTG.

Sequences for miRNA mimics or inhibitors were as follows:

NC mimics: AGCUGCCUACCGAGCUGUGCUC;

miR‐486‐5p mimics: UCCUGUACUGAGCUGCCCCGAG;

NC inhibitors: GCUGUGUACAGGCAGCGACGCA;

miR‐486‐5p inhibitors: CUCGGGGCAGCUCAGUACAGGA.

### 2.4 Quantitative real‐time polymerase chain reaction

Total RNA was extracted from SKOV3 and A2780 cells in light of the protocols of TRIzol reagent (Invitrogen). For the sake of examining gene expressions, cDNA synthesis was accomplished utilizing PrimeScript™ RT reagent kit (Takara). And then qRT‐PCR was conducted by SYBR Premix Ex Taq II (Takara). In the end, 2^−ΔΔCt^ method was adopted to compute. And GAPDH or U6 served as control. Each procedure in this experiment was repeated in triplicate. The primer sequences used in these experiments were listed as follows:

LINC00857 (forward): 5ʹ‐ATCCAAGGCAGGCCCATTC‐3ʹ; LINC00857 (reverse): 5ʹ‐GGAACTCTTGCGGCCAATTC‐3ʹ; YAP1 (forward): 5ʹ‐CCCTCGTTTTGCCATGAACC‐3ʹ; YAP1 (reverse): 5ʹ‐GCAGCCTCTCCTTCTCCATC‐3ʹ; miR‐486‐5p (forward): 5ʹ‐ATTAGTCCTGTACTGAGCTGC‐3ʹ; miR‐486‐5p (reverse): 5ʹ‐TCCTGTACTGAGCTGCCCCGAG‐3ʹ; GAPDH (forward): 5ʹ‐GGAGCGAGATCCCTCCAAAAT‐3ʹ; GAPDH (reverse): 5ʹ‐GGCTGTTGTCATACTTCTCATGG‐3ʹ; U6 (forward): 5ʹ‐CTCGCTTCGGCAGCACA‐3ʹ; U6 (reverse): 5ʹ‐AACGCTTCACGAATTTGCGT‐3ʹ.

### 2.5 Cell counting kit 8 assay

To test cell viability, 10 μL of cell counting kit 8 (CCK‐8) reagent (Beyotime Institute of Biotechnology) was used to incubate transfected SKOV3 and A2780 cells for 2 hours. The absorbance was detected at 450 nm by a microplate reader. Each procedure in this experiment was repeated in triplicate.

### 2.6 Transwell assays

The transfected SKOV3 and A2780 cells (2 × 10^4^) were subjected to re‐suspend in a serum‐free medium and put into the top chamber. Invasion assay was additionally pre‐coated with Matrigel. Then, the lower chamber was filled with 10% FBS. After 24 hours, we utilized methanol to fix migrated or invaded cells and crystal violet to dye. In the end, the number of cells was computed through ×200 microscope (Olympus Corp). Each procedure in this experiment was repeated in triplicate.

### 2.7 5‐ethynyl‐2`‐deoxyuridine staining assay

SKOV3 and A2780 cells were plated in 96‐well plates (5 × 10^4^ cells/well) with EdU medium diluent. Afterward, cells were fixed with 4% paraformaldehyde for half an hour. EdU reagent was produced through RiboBio. Then 4',6‐diamidino‐2‐phenylindole (DAPI) was utilized to stain the nucleus. In the end, a fluorescence microscope (Olympus) was used to observe the proliferative situation. Each procedure in this experiment was repeated in triplicate.

### 2.8 TdT‐mediated dUTP Nick‐End Labeling staining

After transfection, SKOV3 and A2780 cells were fixed with 1% formaldehyde and permeated by Triton X‐100. Following, dUTP‐end labeling from Clontech was utilized to treat cells. DAPI was utilized to stain the nucleus. Finally, cells were watched via fluorescence microscope (NIKON). Each procedure in this experiment was repeated in triplicate.

### 2.9 Flow cytometry analysis

Annexin V‐FITC/PI Apoptosis kit was utilized to evaluate cell apoptosis in line with the protocols of suppliers (BD Biosciences). Cells were collected after being treated with the precooled PBS. Following, they were subjected to double‐stain at least 15 minutes in dark. Finally, it was analyzed through a flow cytometer (BD Biosciences). Each procedure in this experiment was repeated in triplicate.

### 2.10 Glucose uptake, lactate production, and intracellular ATP

18F‐fludeoxyglucose (F‐FDG) uptake experiment was utilized to indicate the intracellular glucose uptake levels of SKOV3 or A2780 cells. The utilization of 12‐well plates was to seed cells for cultivating overnight. And the consistence of each well was 1 × 10^5^ cells. After that, we took out the culture medium and rinsed them by PBS. Then, cells were cultivated in glucose‐free DMEM which included ^18^F‐FDG at RT. One hour later, cells were rinsed and supplemented in 1 mL of 0.5 mol/L NaOH per well for producing cell lysates. Following, a well γ‐counter was applied for detecting the radioactivity of lysates. Besides, the ^18^F‐FDG uptake was radioactive readouts normalized to the number of cells. With regard to lactate production measurements, we gathered cell supernatant for evaluating the lactate concentration. And cell pellets was collected to be lysed and measured ATP level. Each procedure in this experiment was repeated in triplicate.

### 2.11 Subcellular fractionation

In order to isolate the nuclear and cytoplasmic fractions, we utilized the Nuclear/Cytosol Fractionation Kit (BioVision). Quantitative real‐time polymerase chain reaction was conducted to examine the LINC00857 expression in nuclear and cytoplasm fractions. U6 and GAPDH served as nuclear and cytoplasmic control. Each procedure in this experiment was repeated in triplicate.

### 2.12 Fluorescence in situ hybridization

First of all, the fluorescence‐conjugated LINC00857 FISH probe was synthesized by RiboBio. Then, cell specimens were subjected to hybridization with LINC00857 probe. Next, they were stained by DAPI dye and observed by a fluorescence microscope. Each procedure in this experiment was repeated in triplicate.

### 2.13 Luciferase reporter assay

LINC00857 or YAP1 fragment which included miR‐486‐5p target sites (wild‐type and mutant), was separately inserted into the pmirGLO luciferase reporter vector. And we called them as LINC00857‐WT/Mut and YAP1‐WT/Mut. Next, they were subjected to co‐transfection with miR‐486‐5p mimics or NC mimics into SKOV3 and A2780 cells. Forty‐eight hours later, the Luciferase Reporter Assay System (Promega) was utilized to conduct a luciferase experiment. Each procedure in this experiment was repeated in triplicate.

### 2.14 RNA immunoprecipitation assay

First of all, SKOV3 or A2780 cells were lysed in RNA immunoprecipitation (RIP) lysis buffer. Then, cell extract was cultivated with RIP buffer. Following, we supplemented magnetic beads (Invitrogen) which conjugated with anti‐Ago2 and anti‐Immunoglobulin G. Then, they were cultivated with protease K. After digestion, immunoprecipitated RNA was isolated and examined by qRT‐PCR. Each procedure in this experiment was repeated in triplicate.

### 2.15 RNA pull‐down assay

Bio‐miR‐486‐5p and Bio‐NC were composed through Thermo Fisher Scientific. Then, the biotinylated miRNA was subjected to cultivation with cell lysates for one night. Afterward, streptavidin magnetic beads were supplemented. In the end, qRT‐PCR was adopted for evaluating expression levels. Each procedure in this experiment was repeated in triplicate.

### 2.16 Statistical analysis

All of the experiments were carried out independently at least three times. Data were analyzed utilizing GraphPad Prism 7.0 (GraphPad Software), and represented as mean ± SD. Besides, the Student's *t* test or one‐way ANOVA was applied to analyze the group differences. The value of *P* less than .05 indicated statistical significance.

## 3 RESULTS

### 3.1 LINC00857 accelerates cell proliferation, migration, invasion, and glycolysis but restrains cell apoptosis in ovarian cancer

**FIGURE 1:**
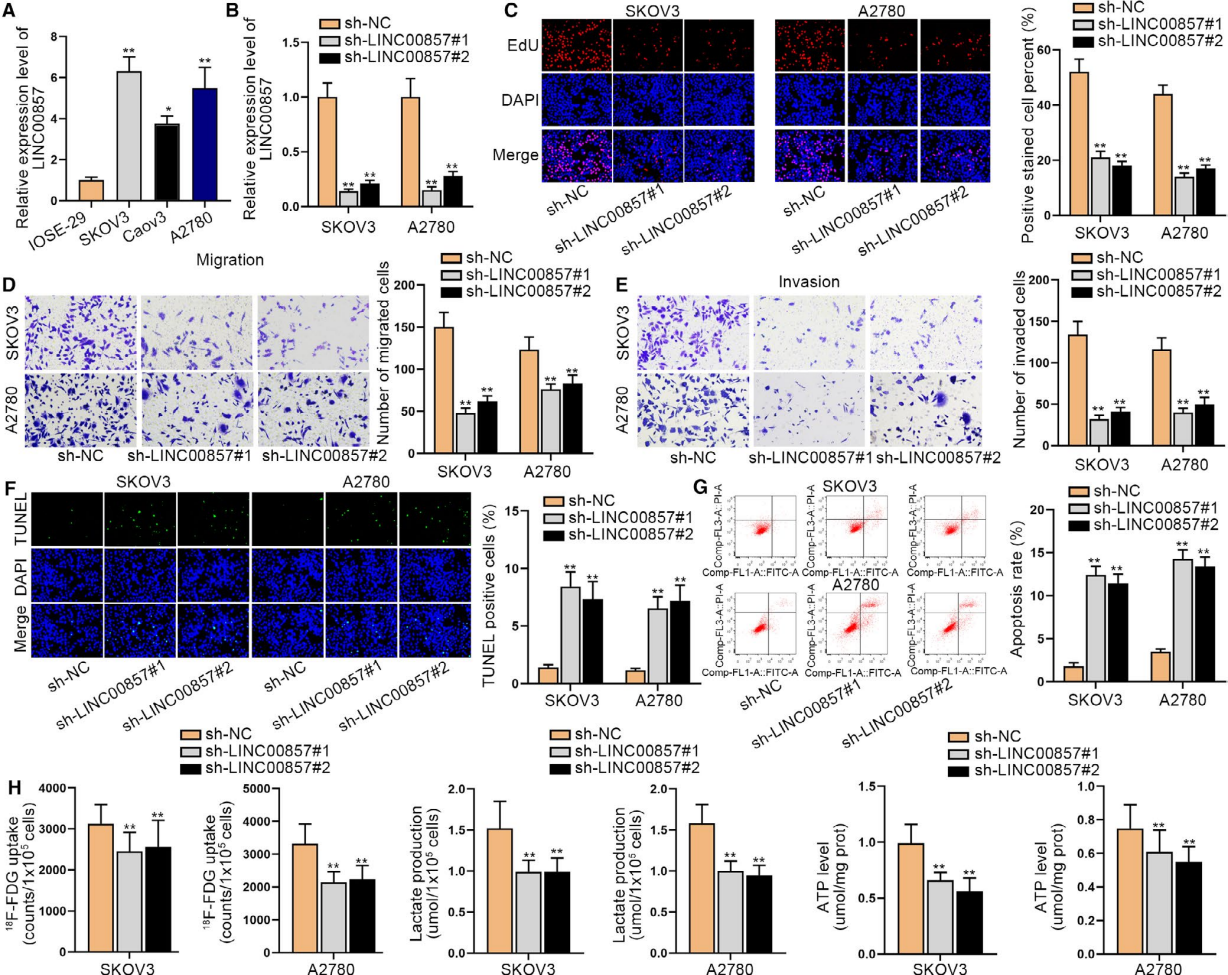
LINC00857 accelerates cell proliferation, migration, invasion, and glycolysis but restrains cell apoptosis in ovarian cancer. A, LINC00857 expression in ovarian cancer was tested through quantitative real‐time polymerase chain reaction (qRT‐PCR). B, The qRT‐PCR assay tested the knockdown efficiency of LINC00857 expression in SKOV3 and A2780 cells. C, EdU assay was utilized to evaluated cell proliferation in SKOV3 and A2780 cells which were transfected sh‐LINC00857. D and E, The migration and invasion capabilities of SKOV3 and A2780 cells were measured by transwell assay when LINC00857 was subjected to knockdown. F and G, TUNEL and cytometry assays were conducted to test cell apoptosis after silencing LINC00857. H, The F‐FDG uptake, lactate release, and ATP production level were detected in SKOV3 and A2780 cells. *P < 0.05, **P < 0.01

Based on the search result of Gene Expression Profiling Interactive Analysis (GEPIA), we determined the high level of LINC00857 in ovarian cancer tissues with the normal ovarian tissues as a negative control (Figure S1A). The upregulation of LINC00857 was further determined in 50 ovarian cancer tissues by comparing their corresponding non‐tumor tissues (Figure S1B). Meanwhile, the expression of LINC00857 in ovarian cancer cells (SKOV3, Caov3, and A2780) was also detected through qRT‐PCR analysis. According to the results, we discovered that LINC00857 was highly expressed in ovarian cancer cells, particularly in SKOV3 and A2780 cells (Figure 1A). Thus, we designed loss‐of‐function experiments in SKOV3 and A2780 cells. Before that, we knocked down LINC00857 in SKOV3 and A2780 cells by transfecting with LINC00857‐specific shRNAs and tested the interference efficiency through qRT‐PCR analysis (Figure 1B). The expression of LINC00857 was exactly reduced after the knockdown. Following, CCK‐8 assay revealed that the cell viability was impaired after the silencing of LINC00857 (Figure S1C). EdU assays indicated that cell proliferation capability was significantly repressed by silenced LINC00857 (Figure 1C). Then transwell assays also displayed that the migration and invasion capabilities of SKOV3 and A2780 cells were hampered when LINC00857 was knocked down (Figure 1D,E). Subsequently, the apoptosis ability was accelerated after the knockdown of LINC00857 (Figure 1F,G). As we all know, glycolysis has a crucial influence on cell proliferation.^19^ Thus, we wanted to know if LINC00857 could regulate the glycolysis process. We detected the F‐FDG uptake, lactate release, and ATP production level in SKOV3 and A2780 cells and the results indicated that the levels of F‐FDG uptake, lactate release, and ATP production were decreased by silenced LINC00857, demonstrating that the knockdown of LINC00857 could restrain glycolysis (Figure 1H). Overall, LINC00857 accelerates cell growth, migration, and glycolysis in ovarian cancer.

### 3.2 LINC00857 inactivates the Hippo signaling pathway and YAP1 acts as an oncogene in ovarian cancer

**FIGURE 2:**
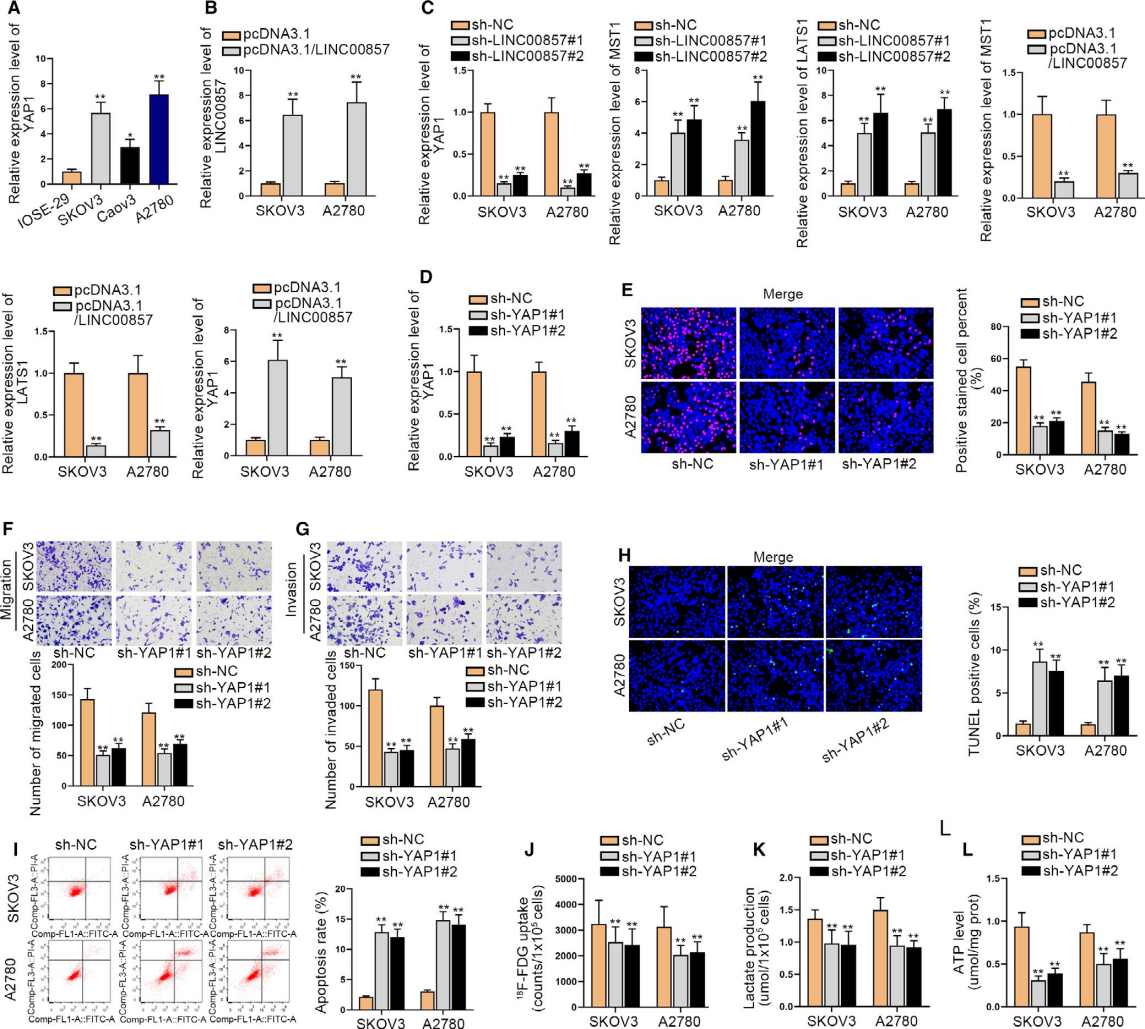
LINC00857 inactivates the Hippo signaling pathway and YAP1 acts as an oncogene in ovarian cancer. A, The expression of YAP1 was detected through quantitative real‐time polymerase chain reaction (qRT‐PCR) in SKOV3 and A2780 cells. B, The qRT‐PCR assay was conducted to test the overexpression efficiency of LINC00857 in SKOV3 and A2780 cells via qRT‐PCR. C, The expressions of YAP1, MST1, and LATS1 in the condition of LINC00857 depletion and LINC00857 overexpression were detected respectively through qRT‐PCR analysis. D, The knockdown efficiency of YAP1 in SKOV3 and A2780 cells was tested by qRT‐PCR. E, Cell proliferation was tested by EdU assays when YAP1 was silenced. F and G, Transwell assays were utilized to measure cell migration and invasion after inhibiting YAP1. H and I, TUNEL and flow cytometry assays were implemented to evaluate cell apoptosis when YAP1 was knocked down. J‐L, The F‐FDG uptake, lactate release, and ATP production level were detected in SKOV3 and A2780 cells after silencing YAP1. *P < 0.05, **P < 0.01

Previous studies have indicated that glycolysis is closely related to the Hippo signaling pathway,^20^ so we speculate whether LINC00857 can regulate the Hippo signaling pathway. Researches have demonstrated that the inactivation of the Hippo pathway can downregulate MST1/LATS1 and upregulate YAP1. By the way, YAP1 is a crucial downstream molecule of the Hippo pathway, which involves in the occurrence and development of malignant tumors.^16^ Here, we designed experiments to evaluate the correlation of LINC00857 and Hippo signaling pathway. First of all, the expression of YAP1 was high in ovarian cancer cells, particularly in SKOV3 and A2780 cells (Figure 2A). Then we overexpressed LINC00857 in SKOV3 and A2780 cells and tested the overexpression efficiency through qRT‐PCR analysis (Figure 2B). LINC00857 expression was elevated in SKOV3 and A2780 cells after transfection. Subsequently, we tested the expressions of YAP1, MST1, and LATS1 in the condition of LINC00857 depletion and LINC00857 overexpression, respectively, through qRT‐PCR analysis (Figure 2C). The results indicated that the expressions of YAP1 were inhibited by LINC00857 depletion but accelerated by LINC00857 overexpression. In contrast, the expressions of MST1 and LATS1 were promoted when LINC00857 was silenced and overexpression of LINC00857 could repress their expressions. Thus, we proved that LINC00857 inactivated the Hippo pathway in ovarian cancer. Next, we began to investigate the functions of YAP1 in ovarian cancer. After we knocked down YAP1 expression in SKOV3 and A2780 cells, EdU assays were conducted to measure the influence of silencing YAP1 on cell proliferation (Figure 2D,E). The results illustrated that YAP1 deficiency led to the notable reduction of cell proliferation. Then through transwell assays, we discovered that cell migration and invasion capabilities were restrained when YAP1 was knocked down (Figure 2F,G). Moreover, it was reflected through TUNEL and flow cytometry assay that cell apoptosis ability could be expedited by YAP1 deficiency (Figure 2H,I). Meanwhile, F‐FDG uptake, lactate release, and ATP production level were also hampered by YAP1 depletion, indicating that the knockdown of YAP1 could repress glycolysis (Figure 2J‐L). Taken together, LINC00857 inactivates the Hippo signaling pathway and YAP1 acts as the oncogene in ovarian cancer.

### 3.3 LINC00857 regulates YAP1 by competitively binding to miR‐486‐5p in ovarian cancer

**FIGURE 3:**
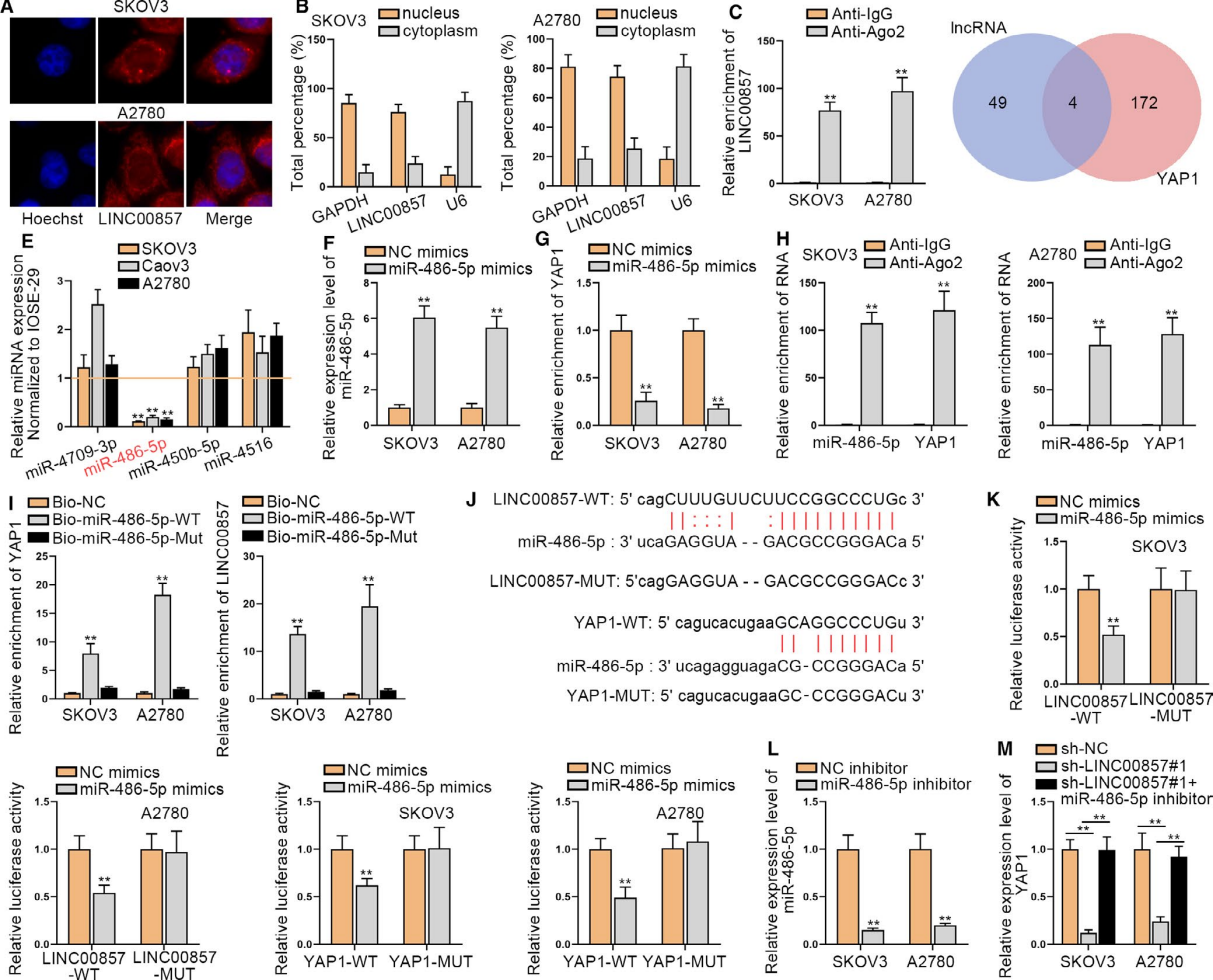
LINC00857 regulates YAP1 by competitively binding to miR‐486‐5p in ovarian cancer. A and B, The distribution of LINC00857 was tested through FISH and subcellular fractionation assays. C, RIP experiment was adopted to detect the enrichment of LINC00857 in Ago2. D, The possible miRNAs were screened out through miRDB. E, The expressions of four miRNAs (miR‐4709‐3p, miR‐486‐5p, miR‐450b‐5p, and miR‐4516) in ovarian cancer cells were detected. F, The overexpression efficiency of miR‐486‐5p in SKOV3 and A2780 cells was tested through quantitative real‐time polymerase chain reaction (qRT‐PCR). G, The expression of YAP1 was tested through qRT‐PCR after overexpressing miR‐486‐5p. H, RIP assay detected the binding situation of YAP1 and miR‐486‐5p. I, RNA pull‐down assay was utilized to prove the combination of LINC00857, YAP1, and miR‐486‐5p. J, The binding sites between LINC00857 and miR‐486‐5p as well as the binding sites between YAP1 and miR‐486‐5p. K, Luciferase reporter assays were utilized to prove the interaction of LINC00857, YAP1, and miR‐486‐5p. L, The knockdown efficiency of miR‐486‐5p was tested through qRT‐PCR. M, The expression of YAP1 was tested when LINC00857 and miR‐486‐5p were inhibited. **P < 0.01

In order to further investigate the regulatory mechanisms of LINC00857 in ovarian cancer, we first tested the distribution of LINC00857 in SKOV3 and A2780 cells through FISH and subcellular fractionation assays (Figure 3A,B). Accordingly, LINC00857 was discovered to take a larger proportion in the cytoplasm of SKOV3 and A2780 cells, which indicated the post‐transcriptional regulation of LINC00857 in ovarian cancer. An increasing number of researches indicated that competing endogenous RNA (ceRNA) network could regulate the progression of cancers. Thus, we conjectured whether LINC00857 could serve as a ceRNA to modulate downstream mRNA by competitively binding to miRNA at post‐transcriptional level. RIP assays further validated that LINC00857 was enriched in Ago2, indicating that LINC00857 may exert the ceRNA function to sponge miRNA (Figure 3C). Then miRDB was utilized to screen out the collective possible miRNAs which had the binding sites with both of LINC00857 and YAP1 (Figure 3D). Four miRNAs were discovered and subjected to qRT‐PCR analysis in ovarian cancer cells for further screening (Figure 3E). Through the results, we determined that miR‐486‐5p presented the lowest level in ovarian cancer cells. Next, miR‐486‐5p was overexpressed by transfecting with miR‐486‐5p mimics in SKOV3 and A2780 cells (Figure 3F). The correlation of miR‐486‐5p and YAP1 was detected through qRT‐PCR analysis. As a result, we discovered that YAP1 expression was declined when miR‐486‐5p was overexpressed (Figure 3G). Then RIP assay demonstrated that YAP1 and miR‐486‐5p were enriched in the Ago2 group, indicating that LINC00857, YAP1, and miR‐486‐5p coexisted in RISC (Figure 3H). Following, RNA pull‐down assays also validated the combination of miR‐486‐5p with YAP1 or LINC00857 (Figure 3I). Using the starBase website (http://starbase.sysu.edu.cn/index.php), we obtained the binding sites of LINC00857 or YAP1 in the miR‐486‐5p seed region (Figure 3J). After that, luciferase reporter assays indicated that the luciferase activities of LINC00857‐WT and YAP1‐WT were hampered in cells with ectopic expression of miR‐486‐5p (Figure 3K). These experiments proved that LINC00857 could sponge miR‐486‐5p which directly targets to YAP1. Then qRT‐PCR analysis was implemented to determine the interference efficiency of miR‐486‐5p (Figure 3L). Meanwhile, we tested the influence of LINC00857 inhibition and miR‐486‐5p on the expression of YAP1 (Figure 3M). We discovered that YAP1 expression could be reduced by LINC00857 deficiency but then recovered by miR‐486‐5p inhibition. Overall, LINC00857 regulates YAP1 by competitively binding to miR‐486‐5p in ovarian cancer.

### 3.4 MiR‐486‐5p restrains cell progression in ovarian cancer

**FIGURE 4:**
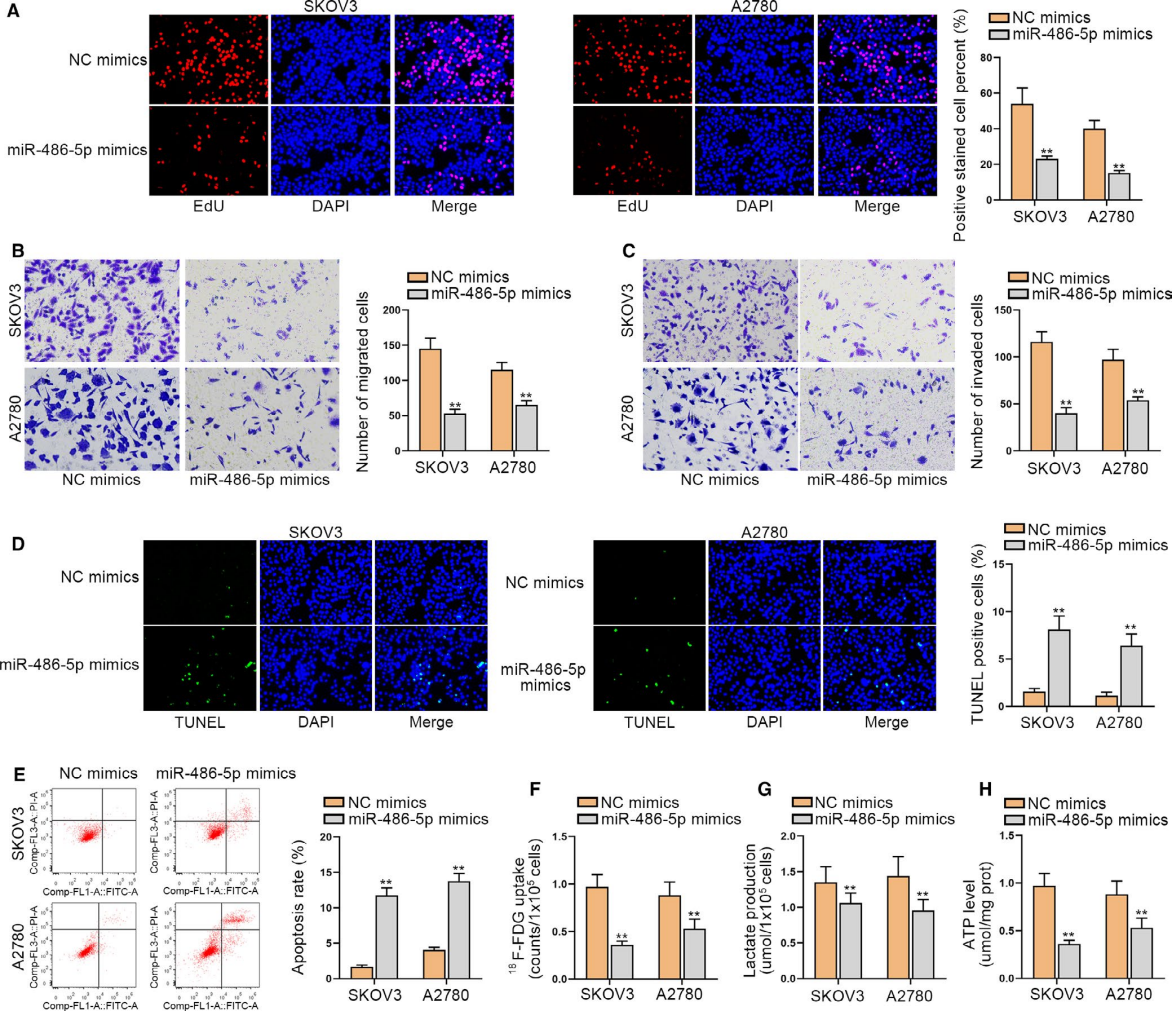
MiR‐486‐5p restrains cellular processes in ovarian cancer. A, Cell proliferation was measured by EdU assays when miR‐486‐5p was overexpressed. B and C, Transwell assays were adopted to estimate cell migration and invasion after overexpressing miR‐486‐5p. D and E, TUNEL and flow cytometry assays were carried out for evaluating cell apoptosis when miR‐486‐5p was upregulated. F‐H, The F‐FDG uptake, lactate release, and ATP production level were detected in SKOV3 and A2780 cells after overexpressing miR‐486‐5p. **P < 0.01

Next, the biological functions of miR‐486‐5p in ovarian cancer cells were investigated. Through EdU assays, we discovered that cell proliferation ability was restrained when miR‐486‐5p was overexpressed (Figure 4A). Then it was demonstrated through transwell assays that cell migration and invasion capabilities were suppressed when miR‐486‐5p was upregulated (Figure 4B,C). It was illustrated by TUNEL and flow cytometry assay that cell apoptosis could be accelerated by miR‐486‐5p overexpression (Figure 4D,E). In addition, F‐FDG uptake, lactate release, and ATP production level were evaluated and we found that they were hampered by the enhanced level of miR‐486‐5p, demonstrating that the upregulation of miR‐486‐5p could repress glycolysis (Figure 4F‐H). In short, miR‐486‐5p restrains cell proliferation and migration, but expedites cell apoptosis in ovarian cancer.

### 3.5 LINC00857 accelerates ovarian cancer progression and glycolysis via regulating YAP1

**FIGURE 5:**
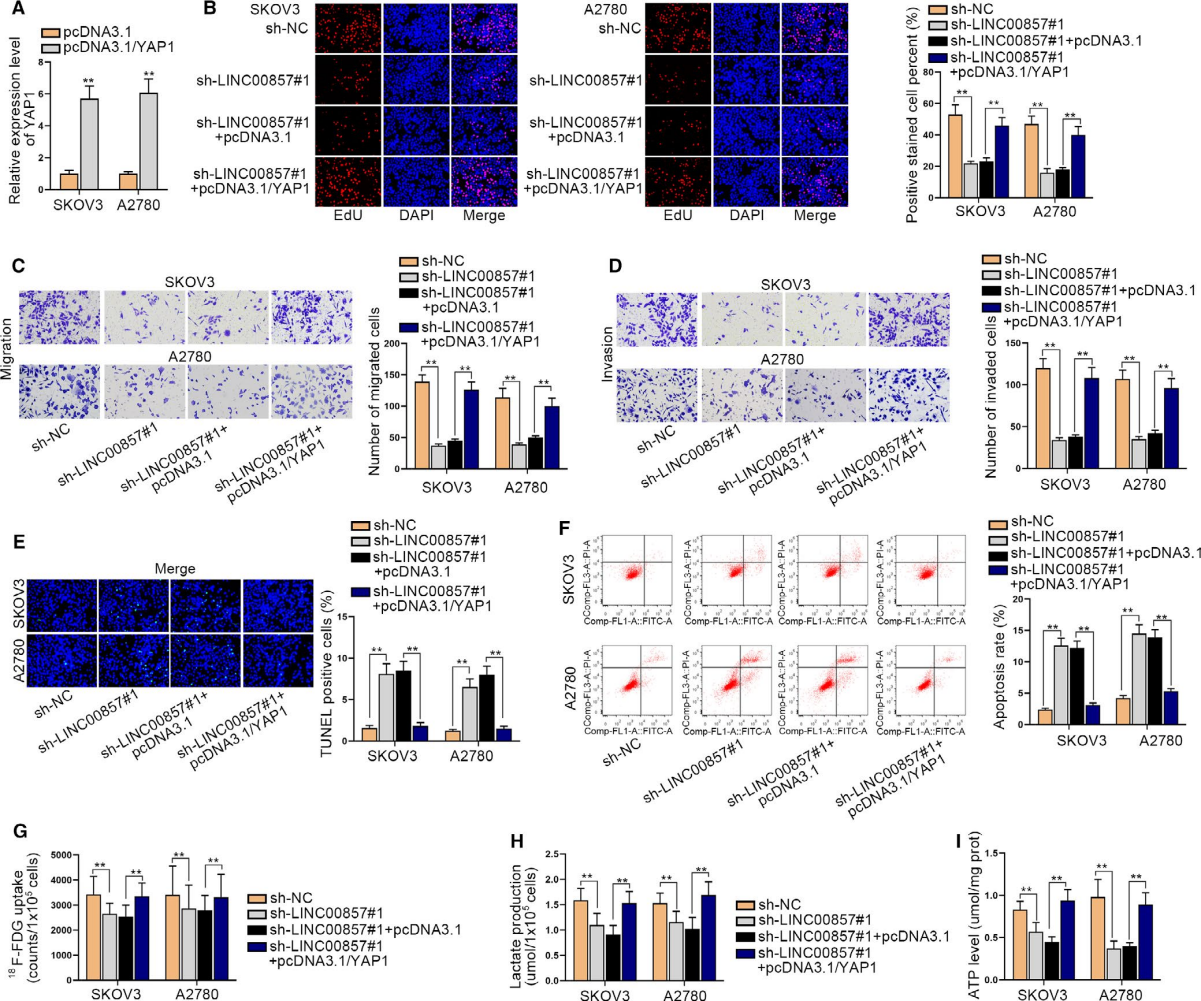
LINC00857 accelerates ovarian cancer progression and glycolysis via regulating YAP1. A, The quantitative real‐time polymerase chain reaction was utilized to test the overexpression efficiency of YAP1 in SKOV3 and A2780 cells. B, Cell proliferation capability was evaluated by EdU assay in transfected cells. C and D, Transwell assays were conducted to evaluate the migration and invasion capabilities of transfected cells. E and F, Cell apoptosis was tested through TUNEL and flow cytometry experiments in different groups (G‐I) The F‐FDG uptake, lactate release, and ATP production level were detected in transfected cells. **P < 0.01

Finally, we probed whether LINC00857 could aggravate ovarian cancer progression and glycolysis via targeting miR‐486‐5p/YAP1 axis through rescue assays. First, we transfected pcDNA3.1/YAP1 into SKOV3 and A2780 cells to elevate YAP1 expression (Figure 5A; Figure S1D). Through EdU assays, we identified that the overexpression of YAP1 counteracted the repressed effect of LINC00857 deficiency on cell proliferation in SKOV3 and A2780 cells (Figure 5B). Following, the transwell assay demonstrated that the upregulation of YAP1 recovered the inhibitory function of LINC00857 depletion on cell migration and invasion (Figure 5C,D). In addition, through TUNEL and flow cytometry assay, we discovered that cell apoptosis ability could be accelerated by silenced LINC00857 but then reversed by overexpressed YAP1 (Figure 5E,F). In the end, it was also illustrated that all of the F‐FDG uptake, lactate release, and ATP production level were declined when LINC00857 was inhibited, but overexpression of YAP1 could offset the inhibitory function. It indicated that the inhibited glycolysis ability could be recovered by the upregulation of YAP1 (Figure 5G‐I). In summary, LINC00857 accelerates ovarian cancer progression and glycolysis via regulating YAP1.

## 4 DISCUSSION

Ovarian cancer is one of the most common female cancers with high morbidity and mortality. So far, its specific pathogenesis has not been studied clearly, so it is necessary to explore the relevant molecular mechanisms in ovarian cancer. An increasing number of researches have indicated that plenty of lncRNAs are discovered to be abnormally expressed in ovarian cancer, and these lncRNAs can serve as ceRNAs. The CeRNA network has been discovered to be a regulatory mechanism in cancer in recent years. It means that lncRNA can sponge miRNA to release the mRNA expression by acting as ceRNA. For example, MLK7‐AS1 regulates the miR‐375/YAP1 axis to promote the progression of ovarian cancer.^21^ LINC00152 expedites cell proliferation by binding with miR‐125b to release MCL‐1 in ovarian cancer.^22^ Moreover, Pro‐transition associated RNA facilitates epithelial‐mesenchymal transition (EMT) progression in ovarian cancer through sequestering miR‐101‐3p to increase the level of ZEB1.^23^ Meanwhile, LINC00857 has been confirmed to exert the function of oncogene in esophageal adenocarcinoma,^12^ lung cancer,^13^ etc. In our research, the expression of LINC00857 in ovarian cancer cells was dramatically upregulated. Function assay demonstrated that LINC00857 depletion effectively restrained cell proliferation, migration, invasion as well as glycolysis in ovarian cancer cells. All of these experiments represented that LINC00857 could accelerate the progression of ovarian cancer.

A flow of researches has indicated that lncRNAs can exert their functions through sponging miRNAs.^24^, ^25^ Previous researches have unmasked that miR‐486‐5p elicits a repressive effect on tumor progression. For example, miR‐486‐5p represses Dock1 and inhibits IL‐22‐induced EMT processes.^26^ MiR‐486‐5p suppresses non‐small cell lung cancer progression.^27^ In our study, bioinformatics prediction and mechanism investigation were implemented and we confirmed that miR‐486‐5p can bind with LINC00857 in ovarian cancer. And miR‐486‐5p restrains cellular processes in ovarian cancer.

Moreover, mechanism assays also proved that YAP1 was the target of miR‐486‐5p. YAP1 confirmed to be an oncogene in some cancers through plenty of researches.^28^, ^29^, ^30^ What is more, YAP1 acted as a crucial downstream gene of the Hippo pathway. Thus, we investigated the correlation between the Hippo pathway and LINC00857. We detected the level of MST1 and LATS1, which were also the important factors in the Hippo pathway, in ovarian cancer cells. And we discovered that the expressions of MST1 and LATS1 were promoted when LINC00857 was silenced and overexpression of LINC00857 could repress their expressions. Thus, we proved that LINC00857 inactivated the Hippo pathway in ovarian cancer. In conclusion, LINC00857 regulates ovarian cancer progression and glycolysis via sponging miR‐486‐5p to upregulate YAP1 by modulating the Hippo signaling pathway, which may provide the new idea for curing ovarian cancer.

## CONFLICTS OF INTEREST

No conflicts of interest exist.

## AUTHORS' CONTRIBUTION

Xueke Lin designed this study and was responsible for article writing and prepared all figures. Dilu Feng and Ping Li collected experimental materials. While Yuchun Lv and Xueke Lin recorded and analyzed the experimental data. All authors have made substantial contributions to this study.

## ETHICAL APPROVAL

The Ethical Approval had obtained from the Ethics Committee of Quanzhou First Hospital Affiliated to Fujian Medical University. Clinical samples were collected and analyzed in accordance with the Declaration of Helsinki. All participants or their guardians had provided the written informed consent.

## Supporting information

Fig S1

Supplementary Materials

## Data Availability

Not applicable.

## References

[1] An YX , Shang YJ , Xu ZW , Zhang QC , Wang Z , Xuan WX et al STAT3‐induced long noncoding RNA LINC00668 promotes migration and invasion of non‐small cell lung cancer via the miR‐193a/KLF7 axis. Biomed Pharmacother. 2019;116:109023. 31150989 10.1016/j.biopha.2019.109023

[2] Torre LA , Bray F , Siegel RL , Ferlay J , Lortet‐Tieulent J , Jemal A . Global cancer statistics, 2012. CA Cancer J Clin. 2015;65(2):87–108. 25651787 10.3322/caac.21262

[3] Bowtell DD , Bohm S , Ahmed AA , Aspuria PJ , Bast Jr RC , Beral V et al Rethinking ovarian cancer II: reducing mortality from high‐grade serous ovarian cancer. Nat Rev Cancer. 2015;15(11):668–79. 26493647 10.1038/nrc4019 PMC4892184

[4] Narod S . Can advanced‐stage ovarian cancer be cured? Nature Rev Clin Oncol. 2016;13(4):255–61. 26787282 10.1038/nrclinonc.2015.224

[5] Clarke‐Pearson DL . Clinical practice. Screening for ovarian cancer. N Engl J Med. 2009;361(2):170–7. 19587342 10.1056/NEJMcp0901926

[6] Sundar S , Neal RD , Kehoe S . Diagnosis of ovarian cancer. BMJ. 2015;351:h4443. 26328593 10.1136/bmj.h4443

[7] Mercer TR , Dinger ME , Mattick JS . Long non‐coding RNAs: insights into functions. Nat Rev Genet. 2009;10(3):155–9. 19188922 10.1038/nrg2521

[8] Schmitt AM , Chang HY . Long noncoding RNAs in cancer pathways. Cancer Cell. 2016;29(4):452–63. 27070700 10.1016/j.ccell.2016.03.010 PMC4831138

[9] Sayad A , Hajifathali A , Hamidieh AA , Esfandi F , Taheri M . Fas‐antisense long noncoding RNA and acute myeloid leukemia: is there any relation? Asian Pac J Cancer Prev. 2018;19(1):45–8. 10.22034/APJCP.2018.19.1.45 PMC5844635 29373891

[10] Chen W , You J , Zheng Q , Zhu YY . Downregulation of lncRNA OGFRP1 inhibits hepatocellular carcinoma progression by AKT/mTOR and Wnt/beta‐catenin signaling pathways. Cancer Manag Res. 2018;10:1817–26. 29997441 10.2147/CMAR.S164911 PMC6033083

[11] Min X , Liu K , Zhu H , Zhang J . Long noncoding RNA LINC003121 inhibits proliferation and invasion of thyroid cancer cells by suppression of the phosphatidylinositol‐3‐kinase (PI3K)/Akt signaling pathway. Med Sci Monit. 2018;24:4592–601. 29969438 10.12659/MSM.908652 PMC6063135

[12] Su W , Wang L , Niu F , Zou L , Guo C , Wang Z et al LINC00857 knockdown inhibits cell proliferation and induces apoptosis via involving STAT3 and MET oncogenic proteins in esophageal adenocarcinoma. Aging. 2019;11(9):2812–21. 31085800 10.18632/aging.101953 PMC6535059

[13] Wang L , He Y , Liu W , Bai S , Xiao L , Zhang J et al Non‐coding RNA LINC00857 is predictive of poor patient survival and promotes tumor progression via cell cycle regulation in lung cancer. Oncotarget. 2016;7(10):11487–99. 26862852 10.18632/oncotarget.7203 PMC4905488

[14] Mo JS , Park HW , Guan KL . The Hippo signaling pathway in stem cell biology and cancer. EMBO Rep. 2014;15(6):642–56. 24825474 10.15252/embr.201438638 PMC4197875

[15] Yu FX , Zhao B , Guan KL . Hippo pathway in organ size control, tissue homeostasis, and cancer. Cell. 2015;163(4):811–28. 26544935 10.1016/j.cell.2015.10.044 PMC4638384

[16] Wu DM , Wang S , Wen X , Han XR , Wang YJ , Shen M et al LncRNA SNHG15 acts as a ceRNA to regulate YAP1‐Hippo signaling pathway by sponging miR‐200a‐3p in papillary thyroid carcinoma. Cell Death Dis. 2018;9(10):947. 30237435 10.1038/s41419-018-0975-1 PMC6148237

[17] Peng X , Ji C , Tan L , Lin S , Zhu Y , Long M et al Long non‐coding RNA TNRC6C‐AS1 promotes methylation of STK4 to inhibit thyroid carcinoma cell apoptosis and autophagy via Hippo signalling pathway. J Cell Mol Med. 2020;24(1):304–16. 31657132 10.1111/jcmm.14728 PMC6933333

[18] Hu G , Dong B , Zhang J , Zhai W , Xie T , Huang B et al The long noncoding RNA HOTAIR activates the Hippo pathway by directly binding to SAV1 in renal cell carcinoma. Oncotarget. 2017;8(35):58654–67. 28938586 10.18632/oncotarget.17414 PMC5601682

[19] Hua Q , Jin M , Mi B , Xu F , Li T , Zhao L et al LINC01123, a c‐Myc‐activated long non‐coding RNA, promotes proliferation and aerobic glycolysis of non‐small cell lung cancer through miR‐199a‐5p/c‐Myc axis. J Hematol Oncol. 2019;12(1):91. 31488218 10.1186/s13045-019-0773-y PMC6728969

[20] Lin C , Xu X . YAP1‐TEAD1‐Glut1 axis dictates the oncogenic phenotypes of breast cancer cells by modulating glycolysis. Biomed Pharmacother. 2017;95:789–94. 28892790 10.1016/j.biopha.2017.08.091

[21] Yan H , Li H , Li P , Li X , Lin J , Zhu L et al Long noncoding RNA MLK7‐AS1 promotes ovarian cancer cells progression by modulating miR‐375/YAP1 axis. J Exp Clin Cancer Res. 2018;37(1):237. 30249278 10.1186/s13046-018-0910-4 PMC6154914

[22] Chen P , Fang X , Xia B , Zhao Y , Li Q , Wu X . Long noncoding RNA LINC00152 promotes cell proliferation through competitively binding endogenous miR‐125b with MCL‐1 by regulating mitochondrial apoptosis pathways in ovarian cancer. Cancer Med. 2018;7(9):4530–41. 30030896 10.1002/cam4.1547 PMC6144155

[23] Liang H , Yu T , Han Y , Jiang H , Wang C , You T et al LncRNA PTAR promotes EMT and invasion‐metastasis in serous ovarian cancer by competitively binding miR‐101‐3p to regulate ZEB1 expression. Mol Cancer. 2018;17(1):119. 30098599 10.1186/s12943-018-0870-5 PMC6087007

[24] Landskroner‐Eiger S , Moneke I , Sessa WC . miRNAs as modulators of angiogenesis. Cold Spring Harb Perspect Med. 2013;3(2):a006643. 23169571 10.1101/cshperspect.a006643 PMC3552340

[25] Eguchi T , Kuboki T . Cellular reprogramming using defined factors and microRNAs. Stem Cells Int. 2016;2016:7530942. 27382371 10.1155/2016/7530942 PMC4921148

[26] Li H , Mou Q , Li P , Yang Z , Wang Z , Niu J et al MiR‐486‐5p inhibits IL‐22‐induced epithelial‐mesenchymal transition of breast cancer cell by repressing Dock1. J Cancer. 2019;10(19):4695–706. 31528235 10.7150/jca.30596 PMC6746125

[27] Tian F , Wang J , Ouyang T , Lu N , Lu J , Shen Y et al MiR‐486‐5p serves as a good biomarker in nonsmall cell lung cancer and suppresses cell growth with the involvement of a target PIK3R1. Front Genet. 2019;10:688. 31402930 10.3389/fgene.2019.00688 PMC6675869

[28] Sekido Y . Targeting the Hippo pathway is a new potential therapeutic modality for malignant mesothelioma. Cancers. 2018;10(4):90. 10.3390/cancers10040090 PMC5923345 29565815

[29] Goto H , Nishio M , To Y , Oishi T , Miyachi Y , Maehama T et al Loss of Mob1a/b in mice results in chondrodysplasia due to YAP1/TAZ‐TEAD‐dependent repression of SOX9. Development. 2018;145(6). 10.1242/dev.159244 29511023

[30] Wu DW , Wang YC , Wang L , Chen CY , Lee H . A low microRNA‐630 expression confers resistance to tyrosine kinase inhibitors in EGFR‐mutated lung adenocarcinomas via miR‐630/YAP1/ERK feedback loop. Theranostics. 2018;8(5):1256–69. 29507618 10.7150/thno.22048 PMC5835934

