# Supplementary figures and images for: LncRNA LINC00857 regulates the progression and glycolysis in ovarian cancer by modulating the Hippo signaling pathway
Source: Cancer Med. 2020 Sep 12;9(21):8122–32. doi: 10.1002/cam4.3322 (PMC7643679; doi:10.1002/cam4.3322)

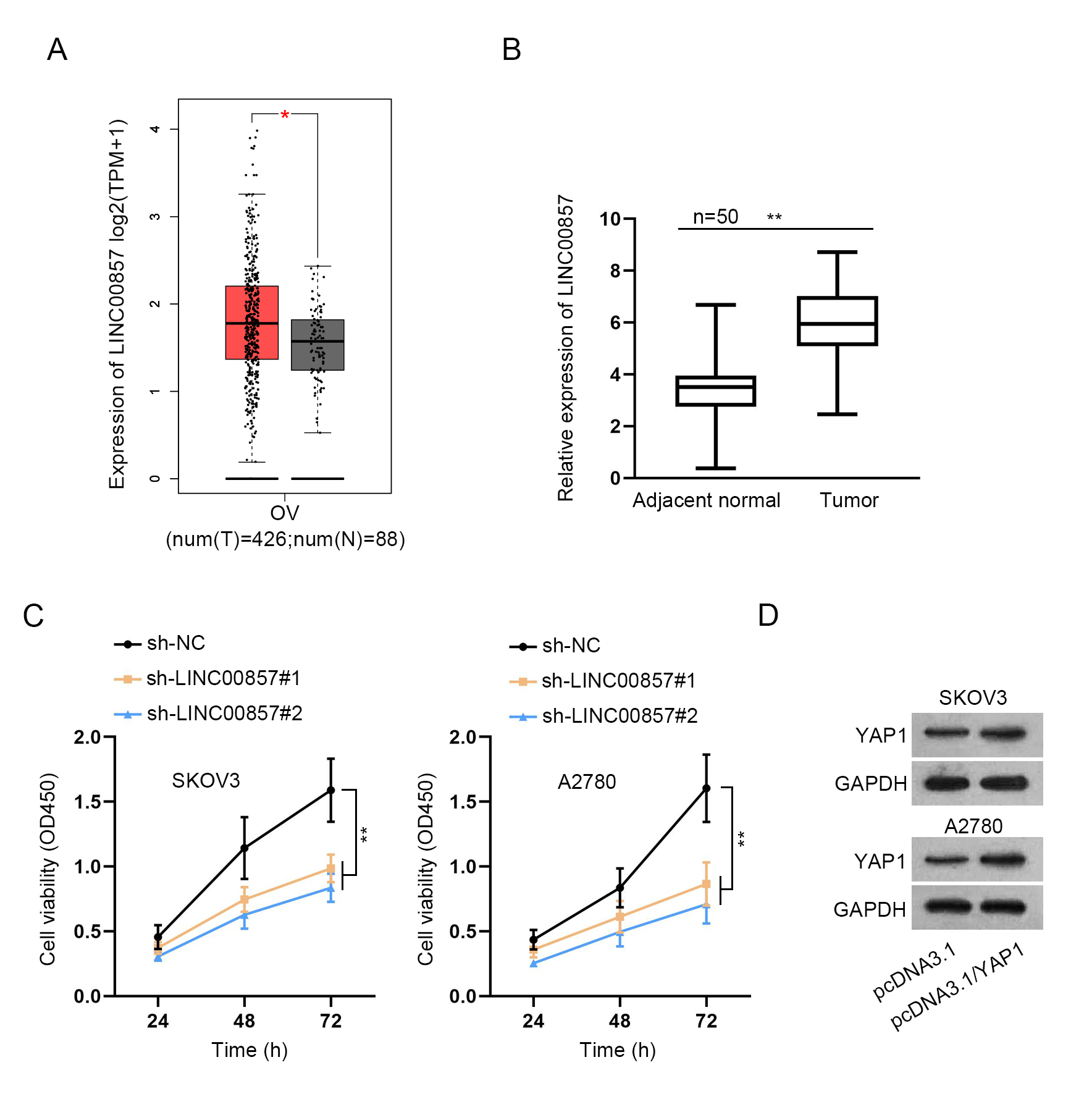

Supplement: Supplementary file 1 — Fig S1 [file CAM4-9-8122-s001.tif]
