# Supplementary material for: LncRNA LINC00857 regulates the progression and glycolysis in ovarian cancer by modulating the Hippo signaling pathway
Source: Cancer Med. 2020 Sep 12;9(21):8122–32. doi: 10.1002/cam4.3322 (PMC7643679; doi:10.1002/cam4.3322)
Supplement: Supplementary file 2 — Supplementary Materials [file CAM4-9-8122-s002.docx]

Figure S1. (A) LINC00857 expression in ovarian cancer tissues and normal tissues was obtained from the GEPIA database. (B) qRT-PCR analysis of LINC00857 expression in 50 pairs of ovarian cancer tissues and adjacent non-tumor tissues. (C) CCK-8 assay was used to measure cell viability in SKOV3 and A2780 cells transfected with sh-NC, sh-LINC00857#1 or sh-LINC00857#2. (D) Western blot analysis of YAP1 protein in SKOV3 and A2780 cells transfected with pcDNA3.1 empty vector and pcDNA3.1/YAP1. **P<0.01.
